# Identification of heart rate dynamics during moderate-to-vigorous treadmill exercise

**DOI:** 10.1186/s12938-015-0112-7

**Published:** 2015-12-21

**Authors:** Kenneth J. Hunt, Simon E. Fankhauser, Jittima Saengsuwan

**Affiliations:** Division of Mechanical Engineering, Department of Engineering and Information Technology, Institute for Rehabilitation and Performance Technology, Bern University of Applied Sciences, 3400 Burgdorf, Switzerland

## Abstract

**Background:**

Heart rate can be used to prescribe exercise intensity for development and maintenance of cardiorespiratory fitness. The aim of this study was to identify the dynamics of heart rate response during moderate-to-vigorous treadmill exercise and to explore parameter dependencies with respect to time, intensity level and step-change direction. The focus was on simple approximate models for subsequent design of heart rate control systems.

**Methods:**

24 healthy, able-bodied male subjects each did two separate, 35-min tests on a treadmill, one at moderate and one at vigorous intensity. Each test had four individual upward and downward steps (1–4). Heart rate responses were modelled as first-order transfer functions with steady-state gain *k* and time constant $\tau$. Models were estimated both for the overall testing periods and for individual step responses within each test.

**Results:**

There were no significant differences in the overall mean values of *k* [24.3 vs. 24.1 bpm/(m/s), $p = 0.88$] and $\tau$ (55.7 vs. 59.5 s, $p = 0.53$) between the two intensity levels. The overall nominal gain for both conditions was $k = 24.2 \pm 8.3$, 21.9–26.6 bpm/(m/s) (mean $\pm$ standard deviation, 95 % confidence interval), and the overall nominal time constant was $\tau = 57.6 \pm 23.6$, 50.9–64.3 s. Analysis of models estimated from the individual steps revealed a significant difference in steady-state gain *k* for upward and downward steps [30.2 vs. 23.6 bpm/(m/s), $p < 0.001$], but no difference in time constant $\tau$ between these two directions (57.5 vs. 54.4 s, $p = 0.52$). For gain *k*, there was no significant main effect of intensity ($p = 0.35$) or intensity–time ($p = 0.86$) interactions, but there was a significant main effect of time ($p < 0.001$). Pairwise comparison with respect to time showed a significant difference between the upward steps at times 1 and 3 [33.0 vs. 27.3 bpm/(m/s), $p < 0.01$], but no significant difference between the downward steps at times 2 and 4 [24.4 vs. 22.8 bpm/(m/s), $p = 1.0$]. For time constant $\tau$, there were no significant main effects of intensity ($p = 0.36$) or time ($p = 0.89$), or intensity–time interactions ($p = 0.23$).

**Conclusions:**

The tight CI-bounds obtained, and the observed parameter dependencies, suggest that the overall nominal model with $k = 24.2$ and $\tau = 57.6$ might serve as the basis for design of a linear time-invariant (LTI) feedback system for real-time control of heart rate. Future work should focus on this hypothesis and on direct comparison of LTI and nonlinear/time-varying control approaches.

## Background

Formal exercise training protocols are implemented for the development and maintenance of cardiorespiratory fitness. The intensity of exercise is commonly prescribed via heart rate (HR), using either a percentage of maximal heart rate or of heart rate reserve (HRR) [1]. HRR is the difference between an individual’s maximal and resting heart rates: $\text {HRR} \triangleq \text {HR}_\mathrm{max} - \text {HR}_\mathrm{rest}$. Current guidelines for healthy adults recommend an exercise duration of 20–60 min, a frequency of 3–5 days/week and an intensity which is categorised to lie between “moderate” and “vigorous” [2]. Moderate intensity is taken to be in the range 40–59 % of HRR and vigorous intensity as 60–89 % of HRR [1] (these intensity bands are adopted in the sequel). It is therefore of relevance to explore the modelling and identification of the dynamics of HR responses, with a view to using such dynamic models for feedback control design, within these two distinct intensity regimes.

Systems for feedback control of HR can be conveniently implemented on cycle ergometers [3] or treadmills [4]; the aim is to automatically set the cycling load or treadmill speed to achieve a prescribed target HR intensity. In such systems, a feedback controller continuously monitors target and measured HR and adjusts the load/speed variable in real time to reduce the HR tracking error. A variety of control design methods have been employed: linear proportional-integral control [5], linear H-infinity control with static nonlinearity compensation [4], combined linear-quadratic and H-infinity optimisation [6], nonlinear output feedback control [7], and a nonlinear neural network approach [8]. Linear, time-invariant (LTI) feedback control of heart rate has also been demonstrated in the context of rehabilitation robotics [9, 10].

In order to design feedback controllers with a desirable level of performance and an acceptable degree of robustness against plant uncertainty, it is necessary to first develop an understanding of the underlying dynamics of the HR response to changes in the manipulated variable. Moreover, it is highly important to investigate any time dependencies or nonlinearities in the responses. Previous research, which is summarised below, has considered some of these sources of plant uncertainty in isolation and usually with a limited sample size. The purpose of the present work was to design an empirical study to systematically investigate these issues in a subject cohort of sufficient size to allow statistically-valid conclusions to be drawn.

In a study with six healthy male subjects doing low-intensity walking exercise on a treadmill, Su et al. [4] showed that satisfactory control of HR could be achieved on the basis of a first-order linear model, albeit with compensation of a static nonlinear model element (a Hammerstein model structure was employed). The same team subsequently observed substantial variability in the model gain and time constant for walking at different speeds, and also a strongly asymmetric step-response behaviour, i.e. different gains and time constants for positive and negative step changes in speed (single-case study, [11]). Asymmetry has also been observed during moderate-intensity treadmill running [12]. A fully nonlinear state-space model has been employed as the basis of HR controllers for low-intensity treadmill walking ([6], six healthy male subjects; [7], two healthy male subjects). In contrast to the studies reviewed above, the present work investigated dynamic responses in subjects running on a treadmill at both moderate and vigorous intensities (40–59 % vs. 60–89 % of HRR, respectively).

The responses of heart rate and oxygen uptake to changes in exercise intensity have been well documented in the exercise-physiology literature [13]. As described by Whipp et al. [14], physiological response kinetics comprise three distinct phases: a rapid and short Phase I response driven by the immediate, and relatively small, cardiodynamic response to exercise onset and lasting $\sim$15 s; a slower Phase II response from about 15 s to 3 min, giving the major increase in the output variable; and then, from about 3 min onwards, a small and usually prolonged Phase III component whose rate of increase is correlated with the work rate level, whereby existence of the Phase III response is dependent upon whether or not the exercise intensity is above the anaerobic threshold. The three phases of the response have each been modelled using mono-exponential functions, i.e. as first-order linear transfer functions, with individual time delays, gains and time constants [15]. Because of the difficulty of reliably estimating the relatively small Phase I component of the response from noisy data, Phase I and Phase II are often combined into a single first-order model whose time constant is referred to as the mean response time (MRT) [13]. The Phase III component, being very slow and lying with the ultra-low-frequency band [16–18], is of little importance in the context of system modelling/identification for feedback design because integral action in any controller serves to eliminate ultra-low-frequency disturbance and uncertainty effects.

The focus in the present work is on the derivation, analysis and interpretation of control-orientated dynamic models, i.e. models which can serve as the basis for the subsequent design of feedback systems for automatic control of heart rate. Within this context, simple approximate models will often suffice as a consequence of the inherent ability of feedback systems to neutralise plant uncertainty [19]. This is in contradistinction to models derived for the purpose of simulation only, where model fidelity is paramount.

Based on the above considerations, the underlying HR dynamics are described here by a first-order linear transfer function to capture the combined Phase I and Phase II kinetics, while very slow (Phase III) components are eliminated by de-trending the measured HR data prior to parameter estimation.

The aim of this study was to identify the dynamics of HR response during moderate-to-vigorous treadmill exercise, thereby investigating variability with respect to time and intensity level, and asymmetry associated with step-change direction.

## Methods

### Ethics and subjects

The study was reviewed and approved by the ethics committee of the Swiss Canton of Bern (*Kantonale Ethikkommission Bern*, Ref. KEK-Nr. 313/14). All subjects were provided with written information about the study and provided their signed consent prior to participation. Formal inclusion criteria were: male, age between 18 and 60 years, able bodied and physically healthy. Exclusion criteria were known cardiovascular, pulmonary or musculoskeletal problems that might have interfered with or contraindicated moderate-to-vigorous treadmill exercise. 24 healthy, able-bodied male subjects were recruited (Table 1) and all subjects completed the study.

All subjects took part in two tests, each carried out on a separate day. Subjects were instructed to avoid: strenuous exercise, alcohol consumption and smoking in the 24 h preceding each test; heavy meals in the 4 h prior to each test; and caffeine in the 12 h period before each test.

**Table 1:** Subject characteristics

Age (years)	27.2 ± 8.9, 18–57
Body mass (kg)	75.0 ± 8.6, 60–98
Height (m)	1.78 ± 0.07, 1.65–1.93
BMI (kg/m^2^)	23.7 ± 2.1, 19.6–27.8

$$n = 24$$, all male

Values are: mean $\pm$ standard deviation, range

*BMI* body mass index (mass/height^2^)

### Test protocol

Target heart rates for moderate and vigorous intensities for each subject were individually set based on HRR, using individual estimates of resting and maximal heart rates. Resting heart rate was measured for each subject at the start of their first test day. Subjects lay resting in a supine position for 5 min. Resting heart rate was then obtained by manual palpation at the radial artery site (wrist). Maximal heart rate was obtained for each subject using the formula $\text {HR}_\mathrm{max} = 220 - \mathrm{age}$ [bpm] (cf. [20]).

**Fig. 1:**
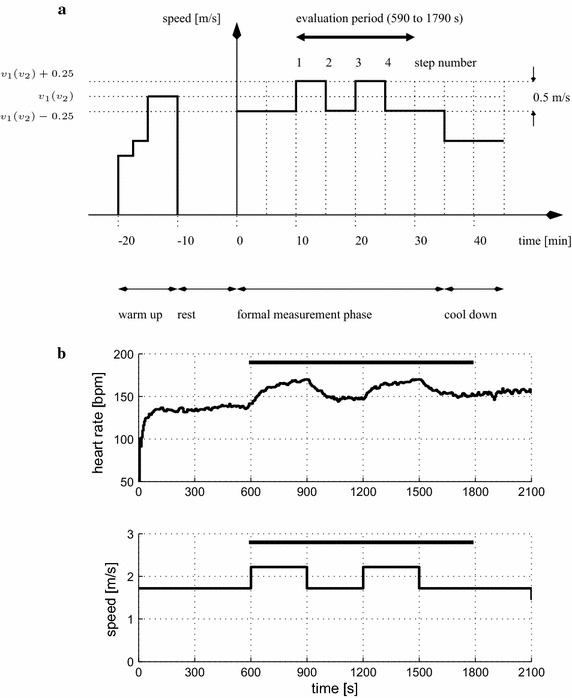
Measurement protocol. **a** Schematic representation. During the warm-up phase, the speed was manually adjusted to achieve the moderate ($v_1$) or vigorous ($v_2$) HR intensity ("Test protocol" section).** b** Example raw data from formal measurement phase (0–35 min), subject S15, vigorous intensity, $v_2=2.0 \,{\rm m}/{\rm s}.$ *Horizontal bars* show the evaluation period for data processing and parameter estimation (590 ≤ *t* ≤ 1790 s)

During the formal measurement phase of the protocol (Fig. 1), subjects ran on a computer-controlled treadmill (model Venus, h/p/cosmos Sports and Medical GmbH, Germany) for 35 min at each of two mean speeds, $v_1$ (moderate) and $v_2$ (vigorous), on different days. The order of presentation of the two test conditions ($v_1$ then $v_2$ vs. $v_2$ then $v_1$) was randomised for each subject. Speed was varied around the mean level $v_1$ or $v_2$ by ±0.25 m/s at 5-min intervals to excite the heart rate dynamics. The 5-min duration for each stage was chosen to ensure the combined Phase I/II response was captured (cf. discussion in “Background” section, [13]). The 35-min measurement phase was preceded by a 10-min warm up and 10-min of rest, and followed by a 10-min cool down period (Fig. 1a).

The treadmill speed was set and heart rate was recorded in real time using Matlab/Simulink (The Mathworks, Inc., USA) running on a PC connected via an RS-232 serial cable to the treadmill. A sample interval of 5 s was used. Raw heart rate was measured directly using a chest belt (model T34, Polar Electro Oy, Finland) and wireless receivers integrated in the treadmill.

The speed levels $v_1$ (moderate) and $v_2$ (vigorous) were established individually for each subject by manual adjustment of the speed during the warm up for each of the two tests. The baseline target heart rate intensity for moderate exercise during the warm up was taken to be 40 % of HRR, i.e. $\mathrm{HR}_1 = \text {HR}_\mathrm{rest} + 0.4\,(\text {HR}_\mathrm{max} - \text {HR}_\mathrm{rest})$, and the baseline target for vigorous exercise during the warm up was set at 60 % of HRR, i.e. $\mathrm{HR}_2 = \text {HR}_\mathrm{rest} + 0.6\,(\text {HR}_\mathrm{max} - \text {HR}_\mathrm{rest})$. The values 40 and 60 % of HRR represent the lower bounds for moderate and vigorous intensity exercise, respectively [1]. The lower bounds were chosen to set $v_1$ and $v_2$ on the expectation that heart rate would drift upwards during the formal 35-min measurement phase due to the possible existence of a Phase III response component, and the desire that the heart rate should not increase beyond the upper bound for each intensity level, viz. 59 or 89 % of HRR [1].

### Nominal model and identification

The dynamic response of heart rate (HR) to changes in treadmill speed (*v*) was modelled as a first-order linear time-invariant transfer function with steady-state gain *k* and time constant $\tau$:

1 $$v \to {\text{HR}}:\;P_{o} (s) = \frac{k}{{\tau s + 1}}.$$

This nominal model is taken to represent deviations around a given mean operating point, i.e. at speeds $v_1$ or $v_2$ and the corresponding mean HR levels, and in the absence of very-low-frequency drift (upward drift of the HR is commonly seen during prolonged moderate or vigorous intensity running, cf. Fig. 1b).

Raw input-output data ($v \rightarrow$ HR), which were recorded with a sample interval $T_s = 5$ s, were processed prior to parameter estimation. First, a range of data of interest was selected: for all data sets, the starting point for estimation of an overall model for each test was just prior to the first upward step in speed at time $t = 590$ s and the end point was selected as 5-min after the final step down in speed at time $t = 1790$ s, $590 \le t \le 1790$ s (cf. Fig. 1b). This selection was to ensure that the same amount of time was spent at the higher and lower speeds, viz. $v_1$ (or $v_2$) +0.25 m/s and $v_1$ (or $v_2$) −0.25 m/s. For the selected data, the HR trend was removed (Matlab ${\tt {detrend}}$ function for linear trend removal) and then the mean levels of the speed and de-trended HR variables were subtracted. Detrending of the HR data serves to eliminate very slow drift corresponding to the Phase III component of the response. Since this drift lies in the ultra-low frequency band, there is no loss of important dynamic information which is available around the bandwidth of model Eq. (1), i.e. at $1/\tau$ rad/s.

To facilitate a sub-analysis of the dynamics of each individual step response, separate models of the form Eq. (1) were estimated for each of the four steps at each intensity level. For this analysis, the selected time ranges were $590 \le t < 890$ s, $890 \le t < 1190$ s, $1190 \le t < 1490$ s and $1490 \le t < 1790$ s (5 min for each individual step response). Before estimation of the individual-model parameters, initial values of speed and HR for each data section were subtracted from the raw data individually over each time range.

For each pre-processed data set, estimates of *k* and $\tau$ in Eq. (1) were obtained using least-squares optimisation (Matlab System Identification Toolbox function ${\tt{procest}}$). Goodness-of-fit was quantified using the normalised root-mean-square error (NRMSE) between the model and measured outputs, denoted as model “fit”, and also by the absolute RMSE.

### Statistics

Statistical analysis was carried out on the identified *k* and $\tau$ parameters to explore whether any differences existed in overall HR dynamics between the moderate and vigorous intensity regimes (paired two-sided t tests).

For the analysis of individual-step dynamics, directional dependence (asymmetry) was explored using a paired two-sided t test on pooled models from all up vs. down steps. For the individual steps, it was investigated whether intensity level ($v_1$ vs. $v_2$) and time (steps 1, 2, 3 and 4) as factors had a significant influence on the dynamics, i.e. whether different dynamics were observed for the four individual step changes in speed at each intensity level, and whether there were significant intensity–time interactions. This was done using two-way repeated-measures ANOVA with intensity and time as independent factors. When significance was found for any factor, Bonferroni correction was used for post-hoc pairwise comparisons.

The significance level was set to 5 %, i.e. $p < 0.05$, for all analyses. Statistical calculations were carried out using the Matlab Statistics and Machine Learning Toolbox (The Mathworks, Inc., USA) and SPSS software (IBM Corp., USA).

## Results

### Test conditions and data processing

For test condition $v_1$-moderate, mean HR during the evaluation period ($590 \le t \le 1790$ s) lay within the individually determined bounds for moderate intensity (i.e. 40–59 % of HRR) in 20 of the 24 subjects. For three subjects, mean HR at $v_1$ was slightly above the 59 % upper limit, but for all three of these subjects mean HR at condition $v_2$-vigorous was substantially higher than for $v_1$. For one subject, mean HR at $v_1$ was slightly below the 40 % lower limit. For the $v_2$-vigorous condition, mean HR in the evaluation time range was within the vigorous-intensity bounds for all 24 subjects.

**Fig. 2:**
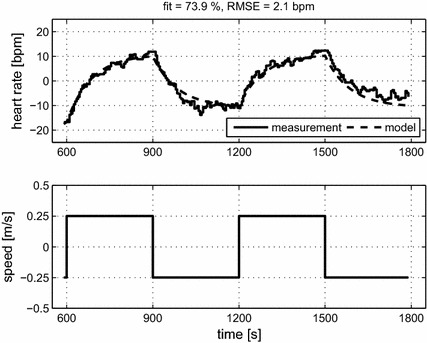
Pre-processed measurement data and model simulation for subject S15: vigorous intensity, $v_2 = 2.0$ m/s; fit = 73.9 %; RMSE = 2.1 bpm. The corresponding raw data are shown in Fig. 1b

For illustration of the data processing and parameter estimation procedures, a single result for subject S15 at vigorous intensity $v_2 = 2.0$ m/s is described (cf. Figs. 1b, 2). Prior to parameter estimation, the HR measurement was de-trended, and mean levels were subtracted from HR and speed. For this single measurement, the estimated model was $P_o = \frac{43.0}{85.0\;s + 1}$. Comparison of the HR measurement and the model simulation (Fig. 2, upper graph) gave a fit of 73.9 % and an RMSE of 2.1 bpm.

### Overall dynamics

Analysis of all outcomes for all subjects over the complete evaluation period ($590 \le t \le 1790$ s) showed that there were no significant differences in the mean values of *k* [24.3 vs. 24.1 bpm/(m/s), $p = 0.88$] and $\tau$ (55.7 vs. 59.5 s, $p = 0.53$) between the moderate ($v_1$) and vigorous ($v_2$) intensity levels (Table 2).

**Table 2:** Outcome measures for model identification at two intensity levels: $v_1$ (moderate) and $v_2$ (vigorous); *p* values for comparison of means for *k* and $\tau$

	Exercise intensity		*p* value
	$v_1$ (moderate)	$v_2$ (vigorous)	
$P_o$	$24.3/(55.7\;s + 1)$	$24.1/(59.5\;s + 1)$	
*k* [bpm/(m/s)]	24.3 ± 9.2, 20.7–28.0	24.1 ± 7.6, 21.1–27.1	0.88 (n.s.)
$\tau$ (s)	55.7 ± 29.9, 43.8–67.7	59.5 ± 15.2, 53.4–65.6	0.53 (n.s.)
Fit (%)	49.6 ± 11.1	54.7 ± 14.2	
RMSE (bpm)	2.8 ± 0.7	2.5 ± 1.1	

Values for *k* and $\tau$ are: mean $\pm$ standard deviation, 95 % confidence interval for the mean

Values for fit and RMSE are: mean $\pm$ standard deviation

$$n =24$$

*p* values are for paired two-sided t tests

*RMSE* root-mean-square error, *bpm* beats per minute, *n.s.* not significant

Since there were no differences between the two intensity levels, an overall nominal model valid across the moderate-to-vigorous intensity regimes, i.e. 40–89 % of HRR, can be obtained as the mean of all 48 measurements (24 subjects $\times$ 2 intensity levels). The overall gain was $k = 24.2 \pm 8.3$, 21.9–26.6 bpm/(m/s) (mean $\pm$ standard deviation, 95 % confidence interval), and the overall time constant was $\tau = 57.6 \pm 23.6$, 50.9–64.3 s. This gives an overall nominal transfer function

2 $$v \to {\text{HR}}:\;P_{o} (s) = \frac{{24.2}}{{57.6s + 1}}.$$

**Fig. 3:**
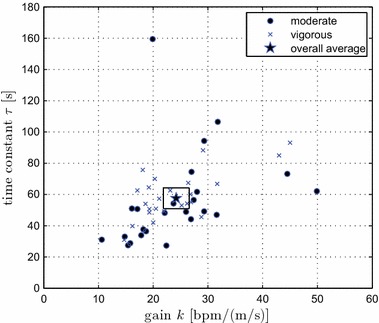
Spread of estimated *k* and $\tau$ values for $v_1$-moderate (*circles*) and $v_2$-vigorous (*crosses*) conditions. The *star* depicts the overall mean values [the overall nominal model, Eq. (2)] for all 48 measurements. The *rectangular box* bounds the 95 % confidence intervals for the overall means of *k* and $\tau$

Dispersion of all estimated *k* and $\tau$ values, together with overall mean *k* and $\tau$ and their 95 % confidence intervals, can be conveniently visualised (Fig. 3).

### Individual steps

Individual dynamic models were identified for each of the four step-changes in speed at both intensity levels. Subject 13 was excluded from this analysis because the data for the 4th step at $v_1$ were poor and the identified *k* and $\tau$ values were sufficiently abnormal to be regarded as outliers. The analysis which follows therefore has $n = 23$.

Possible direction dependencies (asymmetry) in the step responses were analysed by pooling *k* and $\tau$ estimates for all four upwards steps (steps 1 and 3 at intensities $v_1$ and $v_2$) and all four downward steps (steps 2 and 4, levels $v_1$ and $v_2$). This revealed a significant difference in steady-state gain *k* for upward and downward steps [30.2 vs. 23.6 bpm/(m/s), $p < 0.001$, paired two-sided t test], but no difference in time constant $\tau$ between these two directions (57.5 vs. 54.4 s, $p = 0.52$).

**Fig. 4:**
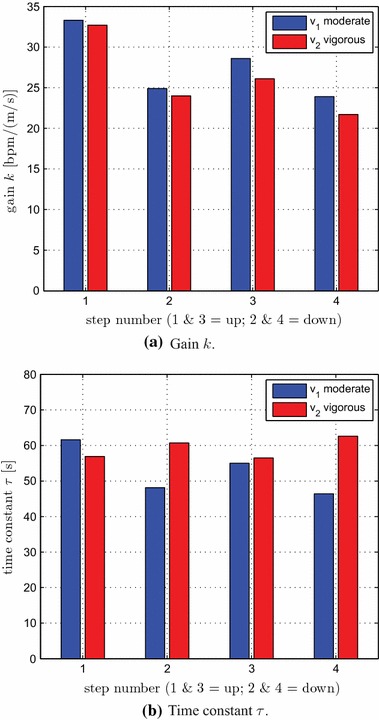
Gain and time constant estimates for individual steps at the two intensity levels $v_1$-moderate (*blue*) and $v_2$-vigorous (*red*). The corresponding numerical values are given in Table 3

The estimated *k* and $\tau$ values for all 8 individual steps (4 at $v_1$, 4 at $v_2$; cf. Table 3; Fig. 4) were analysed separately for possible dependence on intensity level ($v_1$ vs. $v_2$) and time (steps 1, 2, 3 and 4), and for intensity–time interactions. This was done using two-way repeated-measures ANOVA with intensity and time as independent factors. For gain *k*, there was no significant main effect of intensity ($p = 0.35$) or intensity–time ($p = 0.86$) interactions, but there was significant main effect of time ($p < 0.001$). Proceeding to pairwise comparison with respect to time, there was a significant difference between the upward steps at times 1 and 3 [33.0 vs. 27.3 bpm/(m/s), $p < 0.01$], but no significant difference between the downward steps at times 2 and 4 [24.4 vs. 22.8 bpm/(m/s), $p = 1.0$]. For time constant $\tau$, there were no significant main effects of intensity ($p = 0.36$) or time ($p = 0.89$), or intensity–time interactions ($p = 0.23$).

**Table 3:** Gain and time constant estimates for individual steps at the two intensity levels $v_1$-moderate ($k_1$ and $\tau _1$) and $v_2$-vigorous ($k_2$ and $\tau _2$)

	Step number			
	1	2	3	4
$k_1$ [bpm/(m/s)]	33.3 ± 14.0	24.9 ± 11.2	28.6 ± 11.6	23.9 ± 11.6
$k_2$ [bpm/(m/s)]	32.7 ± 6.9	24.0 ± 7.9	26.1 ± 6.4	21.7 ± 8.8
$\tau _1$ (s)	61.6 ± 43.7	48.1 ± 37.0	55.0 ± 30.8	46.4 ± 30.4
$\tau _2$ (s)	56.9 ± 24.0	60.7 ± 34.1	56.5 ± 21.5	62.6 ± 45.4

The values are shown graphically in Fig. 4

$$n =23$$

Steps 1 and 3: up; steps 2 and 4: down

Values for *k* and $\tau$ are: mean $\pm$ standard deviation

## Discussion

The aim of this study was to identify the dynamics of HR response during moderate-to-vigorous treadmill exercise, thereby investigating variability with respect to time and intensity level, and asymmetry associated with step-change direction.

With regard to intensity level (moderate vs. vigorous), analysis of overall models obtained over the entire evaluation period and of individual models obtained from each of the steps showed no significant differences in either steady-state gain *k* or time constant $\tau$ for the two intensities tested. This observation leads to the proposition of a single nominal model obtained by averaging the overall models from all 24 subjects and 2 intensity levels, viz. Eq. (2):

$$v \to {\text{HR}}:\;P_{o} (s) = \frac{{24.2}}{{57.6s + 1}}.$$

Although the individual model parameters appear to be spread widely in the *k*-$\tau$ space (Fig. 3), the calculated bounds on *k* and $\tau$ given by the 95 % CIs are quite tight: 21.9–26.6 bpm/(m/s) and 50.9–64.3 s, respectively. These CIs, which give interval estimates of the respective population means, provide support for adoption of the overall nominal model (2) in the design of feedback controllers for heart rate (further discussed below).

It was found that the steady-state gain *k* for upward steps was significantly higher than for downward steps, but that the time constant $\tau$ did not differ between these two directions. This finding agrees in part with a previous study which investigated step-response asymmetry using 21 healthy male subjects while running on a treadmill, albeit only moderate intensity (70–77 % of HRmax) was considered [12]. It was observed there that the gain was higher, and that the time constant was lower, for positive step changes, but no information was given as to whether these differences were statistically significant.

Analysis of the models estimated here from individual steps revealed a partial and significant dependence of steady-state gain *k* on time, i.e. the plant gain tended to reduce as time progressed. Time constant $\tau$ was found not to change significantly over time.

**Fig. 5:**
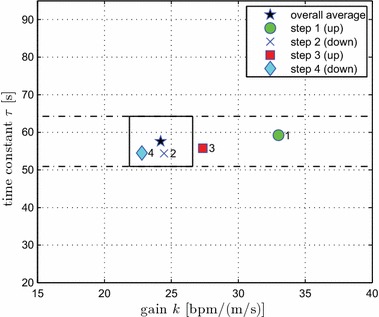
Estimated models for the four individual step changes in speed ($v_1$ and $v_2$ conditions combined, averages over 23 subjects). The *star* depicts the overall nominal model, Eq. (2). The *rectangular box* bounds the 95 % confidence intervals for the overall means of *k* and $\tau$; the *horizontal dash-dot lines* mark the 95 % CI for $\tau$

Visualisation of estimated models for the four individual step changes in speed (with intensity conditions $v_1$ and $v_2$ combined), together with the overall nominal model and its confidence intervals, serves to further illustrate the direction and time dependencies of model parameters (Fig. 5). The time constant $\tau$ for the four individual-step models remains within the confidence interval (CI) for the overall $\tau$. The individual gains *k*, on the other hand, vary substantially and in part lie well outside the 95 % CI for *k* for the overall model. The value of *k* initially lies well above the upper limit of the CI (model 1, first step, upwards), but then converges to within the CI and towards the overall nominal model as time progresses. Thus, the plant steady-state gain has a relatively high value in the initial stages of the exercise (model 1 is from time 10–15 min), but then reduces rapidly towards the overall nominal value with continuing exercise (models 2, 3 and 4 were identified during the periods 15–20, 20–25 and 25–30 min, respectively).

These results lead to the hypothesis that the single model obtained by averaging the overall models from all 24 subjects and 2 intensity levels, viz. Eq. (2) with $k = 24.2$ and $\tau = 57.6$, might serve as the basis for design of a linear time-invariant (LTI) feedback system for real-time control of heart rate. This hypothesis was recently experimentally tested in a separate follow-on study (unpublished data, article in preparation [21]). In that study, an LTI controller was designed using nominal model Eq. (2) and a novel procedure based on shaping of the feedback loop’s input sensitivity function. A total of 30 subjects were tested with this single controller: 20 of these subjects had been included in the present system identification study, and 10 had not. It was found that stable and high-precision control of heart rate was achieved for all 30 subjects, with a mean RMS tracking error of 2.96 bpm. Further, no significant difference was found in the RMSE between the 10 non-identified subjects and 10 matched individuals from the identified group (in fact, RMSE was smaller for the non-identified group, but not significantly so).

Thus, the observation that the dynamics of heart rate response vary between individuals, and in dependence on step direction and time, does not automatically imply that a nonlinear or time-varying feedback system is required for control of heart rate. The robust and high-accuracy LTI results summarised above, [21], were presumably obtained as a consequence of the inherent ability of feedback to neutralise plant uncertainty [19]. Future work should nevertheless focus on whether nonlinear and/or time-varying approaches lead to significantly different performance outcomes by doing studies which directly compare nonlinear/time-varying and LTI approaches within a single subject cohort.

The results and interpretations contained in this study are valid for the moderate-to-vigorous exercise intensity regime, which is the most appropriate range for apparently healthy individuals [1, 2]. Further work is required to ascertain whether HR dynamics are substantially different at very light to light intensities, and also during near maximal exercise. Light intensity is particularly relevant for clinical populations (e.g. patients with cardiac disease), while close-to-maximal exercise is relevant for elite athletes. Identification of the gain and time constant of HR dynamics is clinically relevant as these provide indications of fitness status, adaptations in response to exercise training programmes, and cardiac pathologies.

## Conclusions

The dynamics of HR response during moderate-to-vigorous treadmill exercise displayed no significant dependence on exercise intensity level. Steady-state gain *k* for upward steps was significantly higher than for downward steps, but time constant $\tau$ did not differ between these two directions. Steady-state gain *k* tended to reduce as time progressed, especially in the early stages of exercise, but time constant $\tau$ was found not to change significantly over time.

The tight CI-bounds obtained, and the observed parameter dependencies, suggest that the overall nominal model with $k = 24.2$ and $\tau = 57.6$ might serve as the basis for design of a linear time-invariant (LTI) feedback system for real-time control of heart rate. Future work should focus on this hypothesis and on direct comparison of LTI and nonlinear/time-varying control approaches.
